# *Citrullus colocynthis*-Mediated Green Synthesis of Silver Nanoparticles and Their Antiproliferative Action against Breast Cancer Cells and Bactericidal Roles against Human Pathogens

**DOI:** 10.3390/nano12213781

**Published:** 2022-10-27

**Authors:** Shafqat Rasool, Asima Tayyeb, Muhammad Akram Raza, Hanfa Ashfaq, Sadia Perveen, Zakia Kanwal, Saira Riaz, Shahzad Naseem, Nadeem Abbas, Naushad Ahmad, Suliman Yousef Alomar

**Affiliations:** 1 Centre of Excellence in Solid State Physics, University of the Punjab, Lahore 54590, Pakistan; 2 School of Biological Sciences, University of the Punjab, Lahore 54590, Pakistan; 3 Department of Zoology, Lahore College for Women University, Jail Road, Lahore 54000, Pakistan; 4 Department of Chemistry, University of Leicester, Leicester LE1 7RH, UK; 5 Department of Chemistry, College of Science, King Saud University, Riyadh 11451, Saudi Arabia; 6 Zoology Department, College of Science, King Saud University, Riyadh 11451, Saudi Arabia

**Keywords:** silver nanoparticles, green synthesis, *Citrullus colocynthis*, antibacterial activity, anticancer activity, human breast cancer cell line (MCF7)

## Abstract

The present study investigated the biomedical potential of eco-friendly *Citrullus colocynthis*-mediated silver nanoparticles (Cc-AgNPs). The antibacterial efficacy of Cc-AgNPs was evaluated against two multidrug-resistant pathogenic bacterial strains, *Escherichia coli* and *Pseudomonas aeruginosa*. The antiproliferative and antilipidemic performance of the prepared particles was determined against the MCF7 cell line, a breast cancer cell line. The in vitro antibacterial assay revealed that Cc-AgNPs induced dose-dependent bactericidal activity, as a considerable increase in the zone of inhibition (ZOI) was noted at higher concentrations. Reduced proliferation, migration, spheroid size, and colony formation exhibited the substantial antiproliferative potential of Cc-AgNPs against MCF7 cells. Significant alterations in the expression of cell surface markers, apoptosis, and cell proliferation genes further confirmed the antiproliferative impact of Cc-AgNPs. Moreover, Cc-AgNPs exhibited antilipidemic activity by reducing cellular cholesterol and triglyceride levels and regulating key genes involved in lipogenesis. In conclusion, these results propose that Cc-AgNPs can be employed as a potent tool for future antibacterial and anticancer applications

## 1. Introduction

In the last decade, nanotechnology has emerged as an exponentially developing field with a wide range of applications in the prevention, diagnosis, and treatment of various ailments [1,2]. Nanoparticles, due to their tiny size (1 to 100 nm), tri-layer structure (surface layer, shell layer, and core), ability to encapsulate drugs, and increased surface-to-volume ratio, effectively accumulate at the site of the tumor compared with routinely used drugs. Additionally, nanoparticles also present properties such as better penetration of the cell membrane, circulation longevity, and target specificity [3]. Furthermore, nanoparticles have the highly tunable property of binding to a variety of ligands, which allows them to be effectively used in various biological applications. Many types of nanoparticles, such as metallic (silver, platinum, gold, and palladium), magnetic, ceramic, lipid-based, and polymeric nanoparticles, have been developed [4].

Silver nanoparticles (AgNPs) are widely used to design biomedical devices, wound dressings, and antimicrobial coatings [5]. Chemical precipitation; hydrothermal, biological, and sol-gel processes; and reverse micelle and hydrothermal methods are used for silver nanoparticle synthesis. Green synthesis of AgNPs using plant extracts as a reducing agent is quickly evolving due to the extracts’ nontoxic and cost-effective attributes [6].

*Citrullus colocynthis* (*C. colocynthis*) is a genetically diverse, widespread, and drought-tolerant desert plant belonging to the family Cucurbitaceae. In various studies, the biomedicinal aspects of C. *colocynthis* have been investigated, including its antimicrobial, anticancer, antioxidant, and antilipidemic characteristics [7,8]. It is enriched with various biomolecules, such as flavonoids, glycosides, fatty acids, phenols, and alkaloids [5,9]. These phytochemicals can act as bioreducing and stabilizing moieties in the biosynthesis of AgNPs by converting silver ions (Ag^+^) to free silver. Phytochemical presence on the nanoparticle surface can enhance the functionality of AgNPs as biomedical agents due to their synergetic effects. Furthermore, these bioactive entities can also catalyze redox reactions and serve as stabilizing agents for Cc-AgNP synthesis [6,10].

The aim of the current study was to synthesize Cc-AgNPs using *C. colocynthis* fruit extract (Cc extract) as a reducing agent and investigate the particles’ antibacterial and antiproliferative potential. The results highlight the efficacy of the prepared Cc-AgNPs against *E. coli* and *P. aeruginosa* bacteria and show that they have peculiar antiproliferative properties against the breast cancer cell line MCF7, suggesting that they may play a promising role in future antimicrobial and anticancer therapeutics.

## 2. Materials and Methods

The chemicals used in this work, including silver nitrate (AgNO_3_), tri-sodium citrate (Na3C6H5O7), sodium borohydride (NaBH4), polyvinylpyrrolidone (PVP; Mw: 1,300,000), nutrient broth, auger, and MTT, were of analytical grade and were obtained from commercial sources. Deionized (DI) water was used for solution-making purposes throughout the experiments.

### 2.1. Fruit Extraction and Preparation of AgNPs

**Figure 1:**
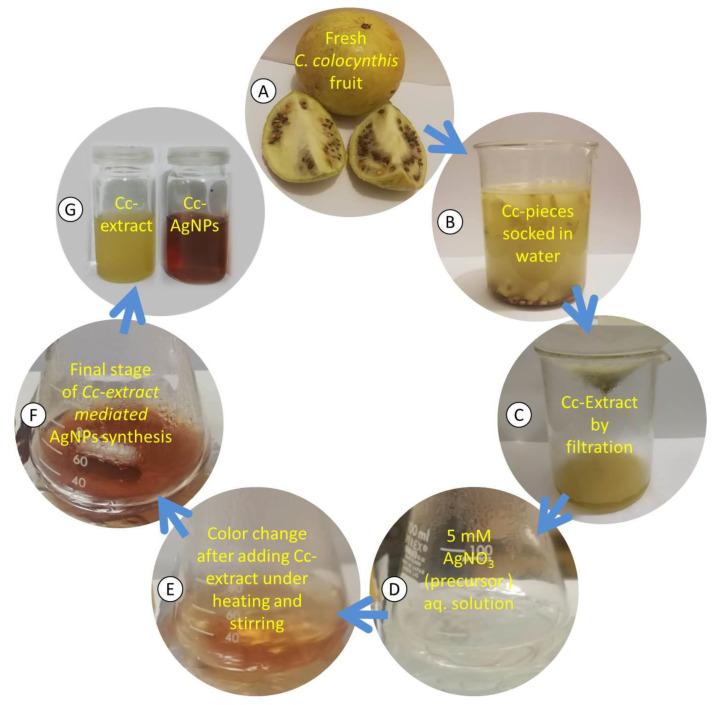
(**A**–**C**) Schematics of different stages during the preparation of *C. colocynthis* extract (Cc extract). (**D**–**F**) Synthesis of *C. colocynthis* silver nanoparticles (Cc-AgNPs) using Cc extract. (**G**) Colloidal samples of Cc extract and Cc-AgNPs.

The aqueous extract of *C. colocynthis* (Cc extract) was prepared by soaking its small pieces in deionized water for three days. The extract was obtained by the filtration of the solution followed by 20 min of centrifugation at 5000 rpm (Figure 1A–C). The preparation of Cc-AgNPs was performed as described by Shawkey et al. [11] with a few modifications. Initially, 20 mL of (5 mM) silver nitrate (AgNO_3_) was heated to a boiling temperature. Then, 2 mL of Cc extract was added to the above solution dropwise with stirring at 200 rpm. The solution color turned from transparent to yellowish brown and then became dark brown within 20 min, showing different phases of the preparation (Figure 1D–G).

### 2.2. Synthesis of AgNPs by Wet Chemical Methods

An already reported wet chemical reduction process was followed for the synthesis of the silver nanoparticles [12]. Briefly, first, tri-sodium citrate (0.5 mL, 30 mM) was dissolved in 50 mL of DI water, and then silver nitrate (1 mL, 5 mM) was added under continual stirring (400 rpm). In the next step, freshly prepared sodium borohydride (0.5 mL, 50 mM) was added at once followed by the addition of PVP (0.5 mL, 1 mM). The reaction was completed in 30 min, producing a bright yellow colloidal solution of chemically synthesized silver nanoparticles (Chem-AgNPs).

### 2.3. Characterization of Biosynthesized Cc-AgNPs

Ultraviolet–visible (UV–Vis) spectroscopy (Shimadzu, UV-1800, Kyoto, Japan) was used to analyze the optical properties of colloidal samples within 300–700 nm wavelengths. The structural nature of the Cc-AgNPs was confirmed by an X-ray diffractometer (XRD) (JSX 3201M, Jeol, Tokyo, Japan), and crystallite size was measured by Scherrer approximation. To identify the attached functional groups on the Cc-AgNPs’ surfaces, Fourier transform infrared (FTIR) spectroscopy (IRTracer-100, Shimadzu, Japan) was performed. Scanning electron microscopy (SEM) and energy-dispersive X-ray spectroscopy (EDX) (FEI Nova NanoSEM 450, Thermo Fisher Scientific, Waltham, *MA*, USA) was used to determine the particle shape, size, and elemental composition. Individual particle morphology was examined by transmission electron microscopy (TEM, Philips CM300ST-FEG, Amsterdam, The Netherlands).

### 2.4. Antibacterial Activity

First, 150 μL of liquid culture of each bacterium (*E. coli* and *P. aeruginosa*) with an optical density of around 1 was spread on nutrient agar plates using sterile cotton swabs, and the plates were labeled accordingly. On each agar plate, 5 wells with 8 mm diameters were bored. Water was used as the control (water); Cc extract as prepared (E = 100%) and three different concentrations (C1: 25 μg/mL, C2: 50 μg/mL, and C3: 100 μg/mL) of AgNO_3_, Chem-AgNPs, and Cc-AgNPs were used for antibacterial activity tests. After overnight incubation at 37 °C, the ZOI around each well was measured.

### 2.5. Cell Culture

MCF7 breast cancer cells were used for all the cell assays. Generally, cells were cultured in DMEM (containing 10% fetal bovine serum and 1% 100 U/mL penicillin/streptomycin) under standard cell culture conditions (37 °C; 5% CO_2_).

### 2.6. MTT Cell Viability Assay

Cells seeded in a 96-well plate were incubated until they reached 70% confluency. A range of working dilutions of Cc extract and Cc-AgNPs were prepared in DMEM, of which 200 µL was added to each well. Control cells were fed with 200 µL of culture media. After 72 h of incubation, cells were washed and incubated with 100 µL of 10% MTT reagent for 3 h. After that, formazan crystals were mixed into 150 μL DMSO/well. The intensity was measured by determining the absorption at 570 nm on a plate reader. The % cell viability was measured as the absorbance of the samples.

### 2.7. Colony Formation Assay

Five hundred cells/well were cultured in six-well plates and incubated in 3 mL of media containing the LC_50_ of Cc-AgNPs. Control cells were cultured in media only. Culture plates were incubated for one week. After that, cells were washed with PBS and fixed in methanol and acetic acid (3:1 ratio) for 15 min. Afterward, staining was performed with crystal violet (0.5%) for 30 min. Cells were washed and photographed through an inverted microscope.

### 2.8. Scratch Assay

Twenty thousand cells/well were seeded in six-well plates. At 95% confluency, cells were washed with PBS, scratched with a micropipette tip, and treated with media containing the LC_50_ dose of Cc-AgNPs. Control cells were cultured in normal culture media. Scratches were periodically examined and photographed, and the gap size was measured up to 72 h.

### 2.9. Spheroid Formation Assay

The spheroid formation assay was performed on 1% agarose-coated culture plates. Ten thousand cells/mL were cultured in media supplemented with the LC_50_ of Cc-AgNPs for 10 days. The morphology and size of spheroids were observed through the Olympus fluorescence microscope software cellSens (Ver.1.18, Wetzlar, Germany).

### 2.10. Determination of Cholesterol and Triglycerides

MCF7 cells treated with the LC_50_ of Cc-AgNPs for 24, 48, and 72 h were collected, and pellets of 1 million cells were obtained. Control group pellets were obtained from cells grown in cell culture media. The total lipids were extracted using a modified Bligh–Dyer method. Lipid extraction was performed using 1 M magnesium chloride and chloroform/methanol (2:1). Lipid pellets were immediately used to determine the cholesterol and triglyceride levels spectrophotometrically from standard calibration curves generated using the standards of known concentrations. For cholesterol determination, the Analyticon Biotechnologies AG 4046 kit and standard cholesterol: SUPLECO were used, while for triglyceride determination, the ABCAM Triglyceride Assay Kit—Quantification ab65336 and standard triglycerides: ab103967 were used.

### 2.11. Gene Expression Analysis

RNA was isolated from cells treated with the LC_50_ of Cc-AgNPs for 24, 48, and 72 h using standard Trizol reagent. Control cells were treated with normal culture media. The quality and quantity of RNA were evaluated with a DeNovix DS-11 FX+ spectrophotometer. RNA (2 µg) was reverse-transcribed by the Thermo Scientific^TM^ RevertAid First Strand cDNA Synthesis Kit (Catalog No. K1622). Gene expression analysis was carried out using Thermo Fisher Sybr Green master mix on the RT-PCR PikoReal qPCR system (Thermo Scientific). GAPDH was used as the housekeeping gene for normalization. The primer sequences of the genes are given in Supplementary Table 1.

### 2.12. Statistical Analysis

Independent *t*-test, one-way, and two-way ANOVA along with Tukey’s post hoc test for multiple comparisons were applied by using Graph Pad Prism 8.4.2 software. Data with a significance level of *p* < 0.05 or lower was considered significant. Results are displayed as means ± standard error of the mean from triplicate experiments.

## 3. Results

### 3.1. UV-Vis Spectroscopy Analysis

**Figure 2:**
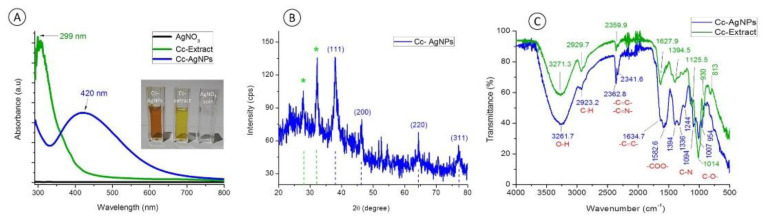
(**A**) UV–Vis spectra of AgNO_3_, *C. colocynthis* extract (Cc extract), and *C. colocynthis* silver nanoparticles (Cc−AgNPs). The inset shows the images of all three colloidal samples. (**B**) XRD pattern of Cc−AgNPs. Characteristic peaks confirmed the FCC crystalline structure of nanoparticles. (**C**) FTIR spectra of Cc extract and Cc−AgNPs show different absorption peaks pertaining to the existence of extract biomolecules on the surfaces of nanoparticles.

The UV–Vis spectra showed the absence of any absorption peak in the entire visible region for the AgNO_3_ sample, confirming the absence of any AgNPs (Figure 2A). The Cc extract showed a narrow peak at around 299 nm, which was attributed to absorption due to biomolecules. These absorptions are related to π → π* transitions, which correspond to the presence of polyphenolic compounds in the extract [13]. In the case of the Cc-AgNPs sample, the 420 nm absorption band is characteristic of AgNPs and is related to surface plasmon resonance (SPR) (Figure 2A). Owing to SPR absorption, nanoparticles in the colloidal form exhibit different colors [14]. The absence of a strong band around 299 nm in the Cc-AgNPs indicated that the extract biomolecules were utilized to reduce the Ag^+^ showing their reducing and stabilizing abilities [11].

### 3.2. XRD Analysis

Six prominent peaks at different locations of 2θ were discerned (Figure 2B). The four characteristic peaks at 38.0°, 46.4°, 64.3°, and 77.1° belonged to reflections from the (111), (200), (220), and (311) planes, respectively (COD ID No. 9013046) [15]. These distinct diffraction planes confirmed that the prepared Cc-AgNPs were metallic in nature with crystalline face-centered cubic (FCC) properties. The peaks at 27.8° and 32.2° (*) can be attributed to the crystallization of bioorganic molecules of the extract on the Cc-AgNPs’ surfaces, serving as capping agents.

### 3.3. FTIR Analysis

The major absorption bands observed for Cc-AgNPs were at 3261, 2923, 2362, 2341, 1634, 1582, 1494, 1336, 1244, 1094, 1007, and 953 cm^−1^. A similar trend in the bands was seen in both the Cc extract and Cc-AgNP spectra, with a noticeable change in intensity and position in the Cc-AgNPs (Figure 2C). The FTIR spectrum signaled the existence of different functional groups, e.g., –O–H, –C–N, –C–O, C–O–H, C=C, and N–O, pertaining to phytochemicals (flavonoids, polyphenols amines, alcohols, aldehydes, amides, and carboxylic acids) [16]. The FTIR of both the Cc extract and Cc-AgNPs indicated that the differences in the shape, intensity, and position of the absorption signals were due to the interaction of extract biomolecules with metallic Ag^+^. Overall, the Cc extract phytochemicals served as bioreducing and stabilizing mediators for Cc-AgNPs synthesis.

### 3.4. Morphological and Compositional Analysis

**Figure 3:**
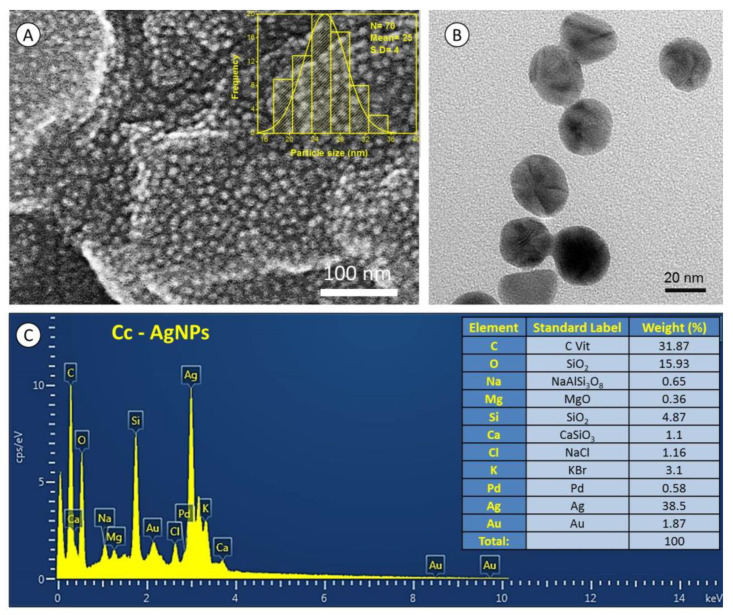
(**A**) SEM micrograph shows a relatively spherical shape of *C. colocynthis* silver nanoparticles (Cc-AgNPs), and the inset represents size distribution histogram. (**B**) TEM image reveals the morphology of individual nanoparticles. (**C**) EDX spectrum of Cc-AgNPs shows strong signal of silver (Ag), and the inset table presents % weight of each element.

SEM showed that Cc-AgNPs were spherical with a size ranging from 17 to 40 nm (Figure 3A). TEM confirmed the SEM results, showing the spherical morphology of individual nanoparticles and a narrow size distribution (Figure 3B). EDX revealed a strong signal (38.5%) at 2.98 KeV [17]. EDX also exhibited some other signals, e.g., Na, C, Ca, O, Mg, Cl, Si, Au, Cl, K, and Pd, with varying intensities (Figure 3C).

### 3.5. Cc-AgNP Treatment Significantly Inhibited the Growth of Pathogenic Bacteria

**Figure 4:**
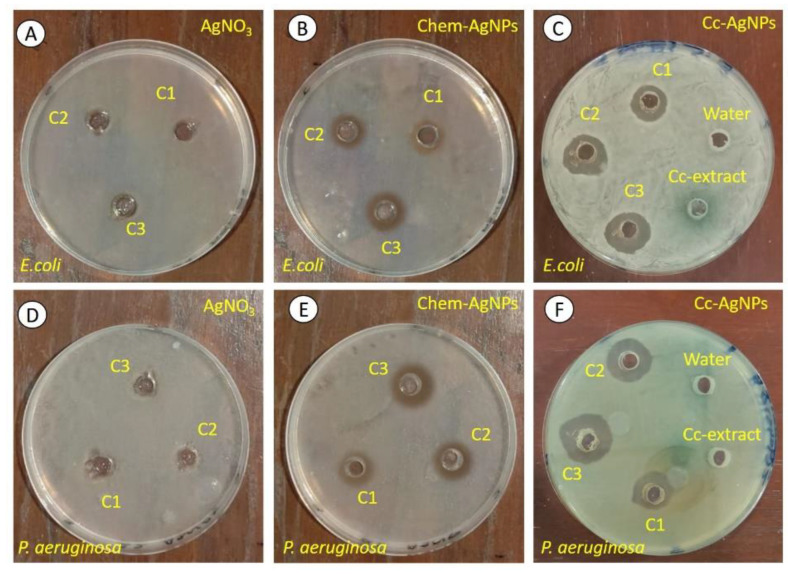
Zone of inhibition (ZOI) showing the bactericidal efficacy of AgNO_3_ (**A**,**D**), Chem-AgNPs (**B**,**E**), and Cc-AgNPs (**C**,**F**) at different concentrations—C1: 25 µg/mL; C2: 50 µg/mL, and C3: 100 µg/mL—against *E. coli* (upper panel) and *P. aeruginosa* (lower panel). Antibacterial activity of DI water and Cc extract against both strains is shown (**C**,**F**).

The Cc extract did not exhibit any bactericidal activity against either bacterial strain (*E. coli* and *P. aeruginosa*). In the case of Cc-AgNPs, concentration-dependent antibacterial activity was observed (Figure 4C,F). The ZOI values for each concentration against *E. coli* were found to be 14.6 ± 0.11 mm (C1), 16.8 ± 0.22 mm (C2), and 17.7 ± 0.34 mm (C3), while those against *P. aeruginosa* were 18.2 ± 0.0 6 mm (C1), 21.4 ± 0.02 mm (C2), and 23.9 ± 0.05 mm (C3) (Figure 4C,D). The comparatively high values of ZOI against *P. aeruginosa* indicated the higher bactericidal potential of Cc-AgNPs against *P. aeruginosa* than *E. coli*. None of the three concentrations of silver nitrate (AgNO_3_) showed any antibacterial efficacy against either strain (Figure 4A,D). However, chemically synthesized AgNPs exhibited concentration-dependent antibacterial behavior against *E. coli* (C1: 11.9 ± 0.21 mm, C2: 12.6 ± 0.43 mm, C3: 15.1 ± 0.26 mm) and *P. aeruginosa* (C1: 12.2 ± 0.15 mm, C2: 15.4 ± 0.09 mm, C3: 17.5 ± 0.11 mm). It was noticed that Cc-AgNPs performed better against both pathogens at all three concentrations.

### 3.6. Reduced Cell Viability, Proliferation, and Growth of MCF7 Cells Treated with Cc-AgNPs

**Figure 5:**
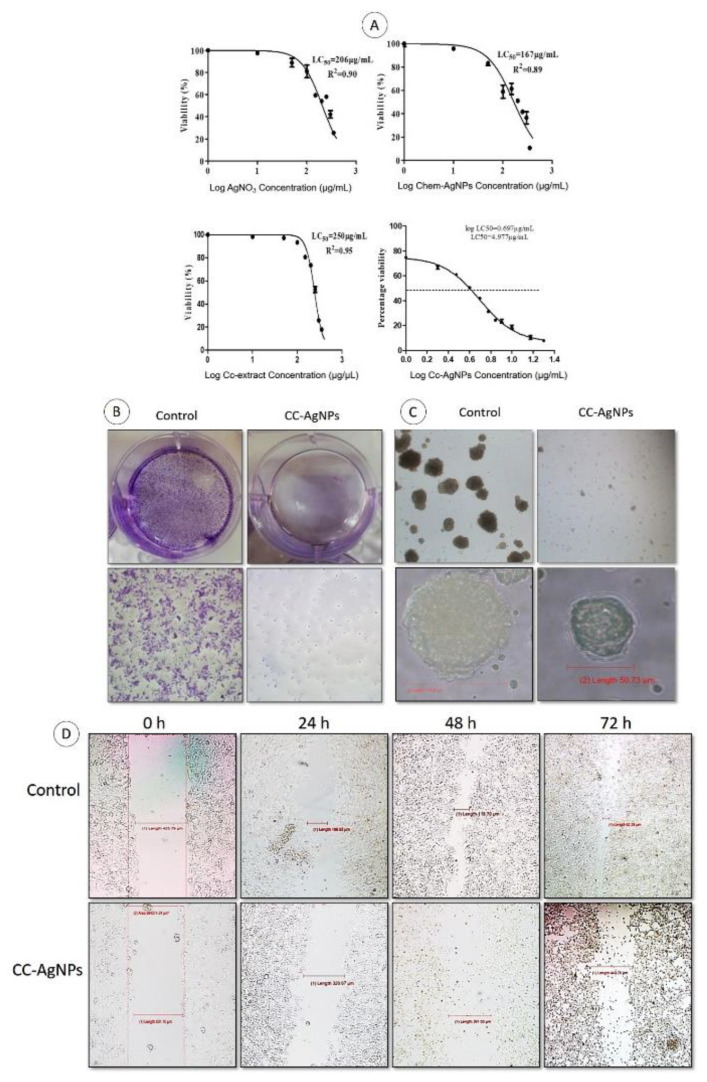
(**A**) MTT cell viability assay of AgNO_3_, Chem-AgNPs, Cc extract, and Cc-AgNPs indicates their dose-dependent cytotoxic potential. (**B**) Colony formation assay indicates the significant potential of Cc-AgNPs to decrease the clonogenicity of MCF7 cells. (**C**) Spheroid formation assay depicts the reduction in 3D growth potential in Cc-AgNP-treated cells. (**D**) Scratch assay shows the reduced migration and proliferation potential of Cc-AgNPs in time-dependent manner.

A dose-dependent cytotoxic effect of Cc-AgNPs was observed (Figure 5A). With an increase in dose, a significant decrease in cell proliferation and viability was recorded. Compared with 206 μg/mL of AgNO_3_, 167 μg/mL of Chem-AgNPs, and 250 μg/mL of native Cc extract, an LC_50_ dose of 5 ± 0.5 μg/mL Cc-AgNPs exhibited a cytotoxic effect that was 50 times greater and substantial antiproliferative potential, despite the significantly lower dose. It was revealed that Cc-AgNPs were potent and significantly inhibited the colony formation ability of MCF7 cells (Figure 5B). Spheroid formation assay further highlighted 3D growth inhibition of cells treated with Cc-AgNPs relative to the control, as shown by the significant decrease in the number and average diameter of spheroids (Figure 5C). The scratch assay indicated a decreased growth and migration potential of Cc-AgNP-treated cells in a time-dependent manner, as depicted by an increase in gap length (Figure 5D).

### 3.7. Regulation of Cell Surface Markers, Apoptosis, and Cell Proliferation Genes in Cc-AgNP-Treated MCF7 Cells

**Figure 6:**
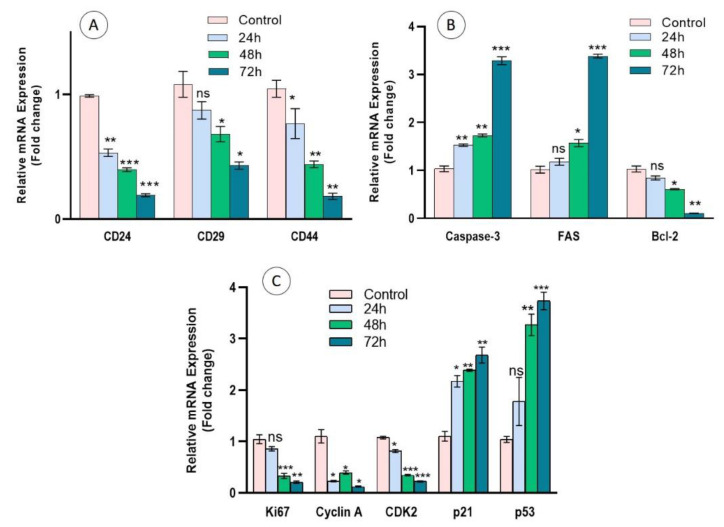
(**A**) Expression of the cell surface markers CD24, CD29, and CD44 was significantly downregulated in MCF7 cells treated with *C. colocynthis* silver nanoparticles (Cc-AgNPs). (**B**) Expression of the pro-apoptotic genes caspase-3 and FAS was upregulated, while the anti-apoptotic gene Bcl-2 showed considerable downregulation. (**C**) Expression of the proliferation marker Ki67, cyclin A, and CDK2 was downregulated, whereas expression of the cyclin/CDK inhibitor p21 and its transcriptional activator p53 was upregulated in time-dependent manner. (* *p* < 0.05, ** *p* < 0.01, *** *p* < 0.001).

The results of the cell proliferation assays were further confirmed by expression analysis of the marker genes involved in proliferation and apoptosis. The expression of the cell surface markers CD-24, CD-29, and CD-44 was significantly (*p* < 0.05) downregulated in a time-dependent manner in Cc-AgNP-treated cells relative to the control (Figure 6A). The relative expression analysis of apoptotic genes indicated considerable (*p* < 0.05) upregulation of the pro-apoptotic genes caspase-3 and FAS and downregulation of the anti-apoptotic marker Bcl-2 in Cc-AgNP-treated cells (Figure 6B). Cc-AgNP treatment also significantly (*p* < 0.05) inhibited the expression of the cell proliferation markers Ki67, Cyclin A, and CDK2. The expression of p21, which inhibits the Cyclin/CDK pathway, and the expression of the tumor suppressor gene p53 were enhanced in Cc-AgNP-treated cells (Figure 6C).

### 3.8. Regulation of Lipid Metabolism by Cc-AgNPs

**Figure 7:**
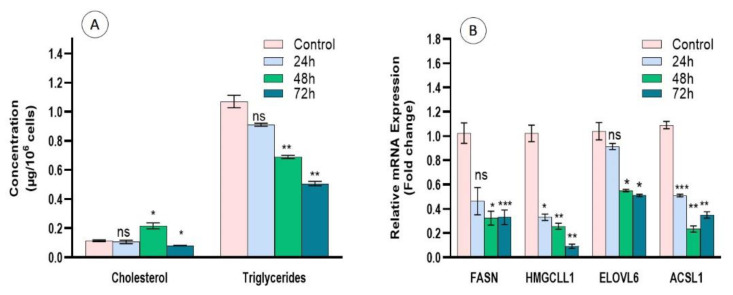
(**A**) Quantification of intracellular cholesterol and triglyceride concentrations in MCF7 cells treated with *C. colocynthis* silver nanoparticles (Cc-AgNPs) in a time-dependent manner after 24, 48, and 72 h treatment compared with control without any treatment. (**B**) Expression of genes involved in lipid metabolism, FASN, HMGCLL1, ELOV6, and ACSL1, after time-dependent treatment with Cc-AgNPs compared with untreated control cells. (* *p* < 0.05, ** *p* < 0.01, *** *p* < 0.001).

Cc-AgNP-treated cells showed a significant decrease in the intracellular cholesterol concentration at 72 h compared with the control. Similarly, the concentration of triglycerides was also significantly decreased at 72 h (Figure 7A). The expression analysis of genes involved in lipid metabolism, FASN, HMGCLL1, ELOVL6, and ACSL1, further strengthened the antilipidemic effect of Cc-AgNPs. Maximum downregulation was shown by HMGCLL1 after 72 h of treatment. FASN was also significantly downregulated at 48 and 72 h. ELOVL6 was also downregulated, with maximum downregulation at 72 h. ACSL-1 showed a significant decrease in expression until 48 h and then increased at 72 h (Figure 7B).

## 4. Discussion

Bioreduced metallic nanoparticles have been recognized for their biocompatible nature along with their many functional properties. They have been considered important in the field of medicine for their greater reactivity and efficiency, which are due to their higher surface-to-volume ratio and quantum effects. AgNPs are important for their antimicrobial and biological activities, and their efficiency may be enhanced by trimming their surfaces with different medicinal and natural biomolecules [18]. The vital role of AgNPs in different biomedical applications is well-established; nevertheless, nanoparticles’ induced toxicity has always been a matter of concern that has limited their applications [19]. The biosynthesis of nanoparticles from different natural materials, including plant extracts, is a proactive approach that can minimize the risks of nanotechnology.

Here, we evaluated C. *colocynthis*-mediated Cc-AgNPs for their cost-effective and eco-friendly use against pathogenic bacteria and the cancerous human MCF7 cells. The findings of characterization techniques, UV–Vis, XRD, FTIR, and EDX analysis confirmed the presence of different Cc extract metabolites on the surface of Cc-AgNPs. In fact, the different bioactive chemical entities, such as flavonoids, amides, aromatic compounds, and amines, of Cc extract served as bioreducing agents for the reduction of Ag^+^ to AgNPs. Furthermore, these bioactive entities catalyzed the redox reactions and worked as stabilizing/capping agents [10,19]. The presence of these phytochemical compounds on the surface of nanoparticles could enhance the functionality of AgNPs as antiproliferative agents via synergistic effects.

Biosynthesized Cc-AgNPs were used to explore their antibacterial and antiproliferative properties. The results showed that pure Cc extract did not show bactericidal activity, which may be due to its aqueous nature [20,21]. Moreover, alcoholic extracts have been found to be more effective than aqueous extracts against various pathogens [22]. Similarly, the non-significant antibacterial results of AgNO_3_ at the tested concentrations against both bacteria were consistent with the literature, as it has been reported that AgNO_3_ exhibits antibacterial activity at higher concentrations [23,24]. The bactericidal performance of Chem-AgNPs was also in line with previously reported findings [12]. The potential bactericidal mechanisms of Cc-AgNPs are nanoparticles’ attachment to and penetration of the bacterial cell wall, disruption of the respiration process, DNA damage by reactive oxygen species production, and apoptosis. The reaction of AgNPs with sulfur- and phosphorus-containing products can disturb the biological activities of bacteria. Thus, the uncontrolled transport of ions due to disturbed permeability, the destruction of DNA due to enhanced oxidative stress, and irregular biological functions make bacterial survival difficult [25].

Previously, *C. colocynthis* has shown potential against different cancer types [6,7,8,26,27]. Cc-AgNPs considerably downregulated the expression of CD24, CD29, and CD44. These markers on cancer cells promote invasion and adhesion and are also responsible for triggering and metastasizing cancer in secondary sites [28,29]. The expression analysis of apoptotic genes showed that Cc-AgNP-treatment activated apoptosis. Cc-AgNPs also reduced the expression of cell proliferation genes. The expression of Ki67, a cell proliferation marker, was downregulated, confirming the diminished proliferation of Cc-AgNP-treated cells. In numerous cancer studies, it has been well-documented that inhibiting the expression of Ki67 is a significant therapeutic approach [30,31,32]. In our study, the downregulation of genes involved in cell cycle progression, cyclin A and CDK2, further indicated that the proliferation and cell division potential of cells was inhibited in Cc-AgNP-treated cells. The Cyclin A/CDK2 complex plays a vital role in cell cycle progression through the S-phase [33]. Targeting this complex is an effective therapeutic strategy against cancer [34,35]. Moreover, the enhanced expression of cyclin/CDK inhibitor, p21, and p53 showed that Cc-AgNP treatment halted cell proliferation. These findings are also in agreement with the previously reported therapeutic strategies against cancer, in which targeting cyclin/CDK inhibitors has been proven to be an effective therapeutic approach [36,37,38].

Cancer cells show entirely different metabolic pathways than normal cells because the metabolic machinery in cancerous cells is reprogrammed to meet their continuous growth and survival demand. The quantity of lipid droplets is higher in cancer cells and supplies a pool of fatty acids to be used for the biogenesis of cellular components and ATP production to sustain the proliferation of cancer cells. A high level of cholesterol and the endogenous synthesis of triglycerides and cholesterol has been observed in breast cancer patients. The expression pattern of genes involved in fatty acid metabolism has been found to be altered in different types of cancer. We observed that Cc-AgNP treatment altered the expression of cholesterol and triglycerides in MCF7 cells. Moreover, the expression of FASN, HMGCLL1, ELOVL6, and ACSL1 was significantly lower in cells treated with Cc-AgNPs. The inhibition of FASN has been reported to suppress the malignancy of human NSCLC cells through the deregulation of glucose metabolism and the AKT/ERK pathway [39]. ELOVL6 was previously identified as a negative clinical predictor of liver cancer, and its knockdown was reported to decrease cancer progression in mice [40]. *HMGCLL1*-IS3 knockdown was found to suppress the proliferation of murine and human CML stem cells. Furthermore, the blockade of HMGCLL1 resulted in G0/G1 cell cycle arrest [41]. ACSLs belong to a group of rate-limiting enzymes in fatty acid metabolism. The knockdown of ACSL1 inhibited the cell cycle, and it suppressed the proliferation and migration of prostate cancer cells in vitro and the growth of prostate xenograft tumors in vivo [42]. Thus, the findings of this study imply that Cc-AgNP targeting of fatty acid metabolism can provide a potential therapeutic strategy against breast cancer progression.

## 5. Conclusions

The synthesis of nanomaterials using different bio-products is a novel and proactive approach that can help to abate some negative aspects of nanotechnology because of the remarkable biological applications of nanomaterials. Cc-AgNPs were prepared using the aqueous extract of *C. colocynthis*, which served as a reducing and stabilizing agent. Investigation of the biomedical applications highlighted the considerable antibacterial potential of Cc-AgNPS against *E. coli* and *P. aeruginosa*. Antiproliferative activity was evidenced by cellular assays, the low expression of cell surface markers, the upregulation of pro-apoptotic genes, the downregulation of proliferation markers, and the upregulation of cyclin/CDK inhibitors in Cc-AgNP-treated cells. Lipid analysis showed that Cc-AgNPs suppressed lipogenesis, a major fuel for cancer growth and proliferation. Further studies with in vivo models and clinical settings will be of the utmost importance to enhance the understanding of the therapeutic potential of Cc-AgNPs.

## Figures and Tables

**Table 1:** List of primers used for this study.

Sr. #	Gene Name	Primer Sequence (5′–3′)	Tm	GC Content	Product Size (bp)
1	CD 24	Forward GTCCAGAAAGGAGAATACAG	56.4	45	277
		Reverse GAAATGGTGCTGGAGATAA	53	42.11	
2	CD29	Forward CAGAGGCTCCAAAGATATAA	54.3	40	213
		Reverse GAGTAAGACAGGTCCATAAG	56.4	45	
3	CD44	Forward GTCCAGAAAGGAGAATACAG	56.4	42.11	332
		Reverse GAAATGGTGCTGGAGATAA	53	45	
4	Caspase- 3	Forward GAACTGGACTGTGGCATTGA	58.4	50	133
		Reverse CCTTTGAATTTCGCCAAGAA	54.3	40	
5	FAS	Forward TGCAGAAGATGTAGATTGTGTGATGA	63.1	50	67
		Reverse GGGTCCGGGTGCAGTTTATT	63.3	50	
6	Ki67	Forward CCTGACAGTGGAAAACCTCT	57	50	219
		Reverse CACAATTTCCTCTGGTGCTG	57	50	
7	Cyclin A	Forward GATGCTGACCCATACCTCAA	57	50	250
		Reverse GGTTGAGGAGAGAAACACCA	57	50	
8	CDK2	Forward ACCAGCTCTTCCGGATCTTT	58	50	186
		Reverse TAGGGTCGTAGTGCAGCATT	58	50	
9	P21	Forward GGAAGACCATGTGGACCTGT	60.5	55	146
		Reverse -GGCGTTTGGAGTGGTAGAAA	58.4	50	
10	P53	Forward GTTCCGAGAGCTGAATGAGG	60.5	55	123
		Reverse TTATGGCGGGAGGTAGACTG	55	60.5	
11	FASN	Forward TTCCGAGATTCCATCCTA	57	44.4	107
		Reverse TGGACGATGTCATCAAAG	57	44.4	
12	HMGCLL1	Forward ATCGACTTTCCCAAACTG	57	44.4	144
		Reverse TAGGAGTAAGGACAGGATAG	57	45	
13	ELOVL6	Forward CTGATCTTCCTGCACTGGTATC	62	50	113
		Reverse TGCACGCCATAGTTCATAGTC	62	47.6	
14	ACSL1	Forward CCTTGCCCAGATGATACTTT	56.8	47.6	88
		Reverse CTCCATGACACAGCATTACA	55.5	47.6	
15	GAPDH	Forward CTCTGATTTGGTCGTATTG	53	42.1	112
		Reverse TGTAAACCATGTAGTTGAGG	54	40	

## Data Availability

Not applicable.
